# Evidence of task-triggered retrieval of the previous response: a binding perspective on response-repetition benefits in task switching

**DOI:** 10.3758/s13423-023-02409-9

**Published:** 2023-11-13

**Authors:** Elena Benini, Malte Möller, Iring Koch, Andrea M. Philipp, Ruyi Qiu, Susanne Mayr

**Affiliations:** 1 Institute of Psychology, RWTH Aachen University, Jägerstr. 17-19, 52066 Aachen, Germany; 2 Psychology and Human-Machine Interaction, University of Passau, Passau, Germany

**Keywords:** Response-repetition effect, Binding and retrieval, Task switching

## Abstract

In task switching, response repetitions (RRs) usually yield performance benefits as compared to response switches, but only when the task also repeats. When the task switches, RR benefits vanish or even turn into costs, yielding an interaction between repeating versus switching the task and the response (the *RR effect*). Different theoretical accounts for this RR effect exist, but, in the present study, we specifically tested a prediction derived from binding and retrieval accounts. These maintain that repeating the task retrieves the previous-trial response, thus causing RR benefits. Retrieval is possible due to the task-response binding formed in the previous trial. We employed a task-switching paradigm with three response options that allowed us to differentiate error types. Across two experiments (*N* = 46 and *N* = 107) we showed that response-repetition errors in response-switch trials were more likely in task repetitions than in task switches, supporting the notion that the previous response is retrieved by the repeating task, despite being wrong. Such a finding is in line with binding and retrieval accounts but cannot be easily accommodated by the competing theoretical accounts. Thus, the present study indicates task-response binding as an important mechanism underlying RR benefits in task repetitions.

## Introduction

In task switching (Rogers & Monsell, 1995; for reviews, see Koch, Poljac, Müller, & Kiesel, 2018; Koch & Kiesel, 2022) participants perform sequences of two or more tasks (e.g., judging the magnitude or the parity of digits via a keypress). In trial *n*, participants perform the same as in trial *n−1* or another task, leading to a task repetition or task switch, respectively. Responses are faster and more accurate in task repetitions than in switches, yielding *task repetition benefits* (Kiesel et al., 2010; Monsell, 2003; Vandierendonck et al., 2010). The correct response in trial *n* may repeat in trial *n−1* or switch, leading to a *response repetition* (RR) or switch, respectively. RRs yield better performance than response switches, but only in task repetitions. In task switches, RR yields *worse* performance than response switches. This interaction constitutes the *RR effect* (e.g., Altmann, 2011; Druey, 2014; Kleinsorge, 1999; Koch et al., 2011).

### Theoretical accounts of the Response-Repetition (RR) effect

One prominent theoretical account of the RR effect assumes a *response inhibition* and a *priming* mechanism (Druey, 2014; Grzyb & Hübner, 2013; Hübner & Druey, 2006). Specifically, responses are inhibited immediately after their execution, preventing potentially erroneous perseveration. Therefore, repeating a response should be more costly than switching it, and RR *costs* are indeed observed in task switches. However, in task *repetitions*, the categorization task (e.g., parity) repeats and, in RR, the *target* category (e.g., even) repeats too. These repetitions, supposedly, strongly prime the *n−1* response, counteracting the effect of the inhibition and yielding RR benefits.

The *reconfiguration account* (Kleinsorge, 1999; Kleinsorge & Heuer, 1999) assumes a hierarchy going from the task to the target category and finally to the response representations. Switching a higher level of the hierarchy commands a switch in all the lower levels. Thus, when either the task or the target category switch, response switches yield benefits compared to RR. RR benefits can thus only emerge when the task *and* the target category repeat.

Rogers and Monsell’s (1995) formulation of an *associative-learning account* suggests that the instructed association between the target category and the response (e.g., “Press left for even”) is strengthened in each respective trial, while the association between this response and the corresponding target category in the other task (e.g., “Press left for larger than five”) is weakened. In task and response repetitions, this strengthening reinforces the relevant association and yields benefits. In RRs but task switches, the strengthening of the now irrelevant association weakens the now relevant association and yields costs (see also Schuch & Koch, 2004). Note that the association between the target category and the response is just one of the possible associations that participants might construe in a task-switching setting. An associative-learning account whereby the *task* becomes associated with the response would make the same predictions concerning the RR effect as the episodic-retrieval accounts that we illustrate in the next paragraph.

The *episodic-retrieval* accounts assume that so-called *bindings* between features are formed in each trial, and can be retrieved when one or more features repeat (e.g., Altmann, 2011; Gade et al., 2014). For example, the Theory of Event Coding (TEC; Hommel et al., 2001) and the Binding and Retrieval in Action Control (BRAC) framework (Frings et al., 2020) explain the RR effect by assuming that task-response bindings^1^ are formed in trial *n−1* and are retrieved in trial *n* when the task and/or the response repeat. In task repetitions, response switches cause costs since the retrieved response interferes with the currently required response. In task switches, repeating the response causes interference since the retrieved task interferes with the current task. When both task and response switch, no *n−1* binding is retrieved. Therefore, repeating the task retrieves the *n−1* response even without a target-category repetition. The three accounts reported earlier, instead, describe RR benefits occurring when the target category repeats.

Yet the exact mechanisms underlying the RR effect remain unclear. In the present study, we tested whether repeating the task retrieves the *n−1* response, which could explain RR benefits in task repetitions. Importantly, we tested response retrieval when the target category switched, thus when the alternative accounts^2^ predict neither retrieval nor priming of the *n−1* response.

### The present study

Across two experiments, we measured the probability of *erroneously* repeating the *n−1* response in a task-switching paradigm with three response alternatives (Koch et al., 2023; Moretti et al., 2023; Steinhauser & Gade, 2015). In *response switches,* this permits distinguishing (1) the correct response, (2) an incorrect response repeating the *n−1* response (a *response-repetition error*), and (3) the remaining response (a *residual error*). If repeating a task retrieves the previous response, as the episodic-retrieval accounts maintain (e.g., Altmann, 2011; Frings et al., 2020), we expected more *response-repetition errors* in task repetitions than task switches (where the task does not retrieve the *n−1* response). A similar error-based approach exists in the negative-priming literature, wherein a repeating distractor was shown to retrieve the *n−1* response (e.g., Mayr & Buchner, 2006).

Importantly, in the response-repetition errors analyses, we exclusively considered response switches, wherein the target category always switches. In target-category switches, none of the competing accounts predicts that the *n−1* response should be reactivated or primed. Hence, only episodic-retrieval accounts predict a higher probability of response-repetition errors in task repetitions than in task switches.

## Experiment 1

### Method

#### Participants

We recruited 48 students at RWTH University who received payment (5 € for about 30 min) or course credits. Participants were between 18 and 35 years old and had a good knowledge of English. The average overall error rate (ER) in the sample was 18.9%, and we removed from the analyses participants who had an average ER greater than 40% since they were outliers. Thus, 40% was used as a threshold for both experiments. This implied removing two participants in Experiment 1. The remaining 46 participants (31 females, one did not declare) were on average 23.8 ± 3.8 years old, and two were left-handed.

Averaging the RR effect, i.e., the interaction of task relation and response relation in previous task-switching studies from our group, we obtained large effect sizes, namely η_p_^2^ = 0.37, Cohen’s *d*_*z*_ = 0.77 (Benini et al., 2023: η_p_^2^ = 0.27, η_p_^2^= 0.17; Benini et al., 2022: η_p_^2^ = 0.42, η_p_^2^= 0.24; Kandalowski et al., 2020: η_p_^2^ = 0.17, η_p_^2^= 0.40; Koch, Frings, & Schuch, 2018: η_p_^2^ = 0.72, η_p_^2^= 0.58). Considering the interaction as a paired-samples two-tailed *t* test on the difference score, according to G*Power (Faul et al., 2009), with 46 participants we had a 99% probability to detect such an effect (i.e., power) with alpha = 0.05. If we weight each effect size by the respective sample size, the average effect size becomes smaller, namely η_p_^2^ = 0.17, Cohen’s *d*_*z*_ = 0.45, but it can still be detected with more than 85% power with 46 participants.

The ethics committee of the Philosophical Faculty at RWTH Aachen University approved the procedure of both Experiments 1 and 2. Neither experiment was preregistered. The data, the analyses, and the experimental materials are available at 10.23668/psycharchives.13033.

#### Stimuli, Tasks and Responses

The targets were the digits from three to eight,^3^ presented at the centre of the screen in Arial black font on a white background. Participants had to categorize the digits as small, medium or large according to their value (small: 3 and 4, medium: 5 and 6, large: 7 and 8) or their font size (small: 30 pt and 42 pt, medium: 67 pt and 93 pt, large: 148 pt and 177 pt).^4^

The value task was cued by a horizontal rectangle (100 × 40 pixels), and the font size task by a vertical rectangle (40 × 100 pixels), or vice versa, counterbalanced between participants. The rectangles were presented in black, 150 pixels above the screen centre. In both tasks, for all participants, the response-key mappings were fixed: the V key indicated “small”, the B “medium” and the N “large”. Participants pressed them with the index, middle and ring fingers respectively.

#### Procedure

We programmed our online experiment with Gorilla Experiment Builder (Anwyl-Irvine et al., 2020), where it was also hosted. Data collection took place between July 2021 and September 2021. Participants could only participate using Chrome or Safari as the browser and a computer or laptop as the device. They were sent an email containing the link to the experiment and they had a maximum of 1 h and 15 min to complete the experiment.

The first experimental screen reminded participants to pick a quiet place and to find a comfortable position. They were invited to open the study in a new window and/or to enter full-screen mode by pressing the F11 key on the keyboard. They could choose to read the informed consent and the data protection regulation in German or English and, after having accepted both, they saw the experiment instructions.

The instructions were in English and explained the two tasks, the cue-task mappings and the response-key mapping. All participants were instructed to respond with the right hand, independent of their handedness. They were shown a picture of a right hand correctly placed on the keyboard and were encouraged to keep their fingers on the respective keys at any time. Such a picture also reminded them of the response-key mapping since on each finger we digitally pasted the words “small”, “medium” and “large”, accordingly. This picture was shown again before they started the training trials and before each new block of trials. The instructions included the digit 3 in all the possible six font sizes one next to the other. Further, we showed the 3 in the six sizes in six sequential screens, each explaining the correct label, for example: “This is an example of a medium font size” (with “medium” printed in bold). Participants could navigate back and forth between the six screens at will. Then, participants saw the vertical and the horizontal cues and the task-cue mapping, which was counterbalanced between participants. They were asked to respond as fast and accurately as possible but to focus especially on speed even though this may result in making some more errors. However, they were instructed to maintain an overall average accuracy above 40%.

There were 36 training trials during which participants were shown each digit six times, three per task, and they saw each font size at least once (but it was not guaranteed that they saw each combination of digit and font size). Each training trial was identical to the experimental trials, but it included accuracy and speed feedback. Accuracy feedback helped participants learn the tasks and the response keys, while speed feedback was aimed at increasing participants’ speed and consequently the probability they made an error. We reasoned that more errors would prevent floor effects in our data. Therefore, the sentence: “Please, try to be faster!” was already presented after 500 ms if no response was given, and remained on the screen for 400 ms. A red cross followed an incorrect response, a green tick followed a correct response.

Each trial started with a fixation cross presented for 1,400 ms, followed by the cue and, after 600 ms, by the target. The cue and the target remained on the screen until the response or for 1000 ms after which they disappeared, although participants could still give their response for another 1000 ms, unbeknownst to them (although they could realise it during the training if they got accuracy feedback to a response in that interval). We reasoned that removing the cue and the target would create an incentive to respond before the relevant information disappeared, thus increasing speed and the probability to make an error. A trial was timed-out if no response was given within 2 s from target onset.

Upon completing the training, participants could decide to read the instructions again, and repeat the training or start with the experimental trials. The experiment consisted of five blocks of 72 trials each, where each digit was presented in all font sizes, once per task. The trial sequences were pseudorandomized with the constraint that there were either 35 or 36 task switches (and thus, either 36 or 35 task repetitions, respectively). We allowed the immediate repetition of a certain font size and/or digit from trial *n−1* to trial *n*, not to bias response probabilities. During a self-paced break between blocks, participants were shown their overall accuracy and were reminded to respond as fast as possible and of the cue-task mapping. Before starting a new block, participants were reminded to place their fingers on the V, B and N keys as shown in the picture presented, and not to move them until the following break. After completing the five blocks, participants filled out a demographic questionnaire, and they were thanked and debriefed through a written text explaining the rationale of the experiment.

#### Design and analyses

The experiment comprised a 2 × 2 within-subject design with the factors *task relation* (repetition vs. switch) and *response relation* (repetition vs. switch). Our main hypothesis involved response-repetition errors only,^5^ and consisted of a larger probability of response-repetition errors in task repetitions than in switches, due to task-triggered retrieval of the previous response. We only considered response switches since exclusively in these trials we could differentiate between response-repetition errors and residual errors. Moreover, among response switches, we considered only errors to test the probability of a response-repetition error *given that an error is made*. Our dependent variable was therefore the probability of response-repetition errors. This was calculated by dividing the number of response-repetition errors in each of the two relevant experimental conditions (task repetitions vs. switches) by the total number of errors in response switches in that condition in the cleaned dataset (see the *Results* section for the dataset cleaning procedure). Thus, our dependent variable is orthogonal to the overall probability of committing an error.^6^ This dependent variable was submitted to a one-way within-subjects ANOVA with task relation (repetition vs. switch) as the within-subject independent variable. Since the only other possible error in response switches is a residual error, the probability of residual errors is complementary to the response-repetition one.

Furthermore, to examine whether our data pattern replicates typical task-switching findings and, specifically, the interaction between task relation and response relation (i.e., the RR effect), we ran two further two-way within-subjects ANOVAs also including RR trials. Thus, task relation (repetition vs. switch) and response relation (repetition vs. switch) were the independent variables, and ERs and correct-trials reaction times (RTs) were the dependent variables.

### Results

#### Probability of RR errors

From the complete dataset, we removed response repetitions (32.1%), the first trial of each block (1.4%), post-error trials (19.9%), all trials with RTs shorter than 200 ms (0.3%) and timed-out trials (0.9%). From the response switches, we thus discarded 21.6% of the original trials. The 78.4% of response switches that we analysed included 82.6% correct responses, 8.5% response-repetition errors, and 8.7% residual errors.

**Fig. 1:**
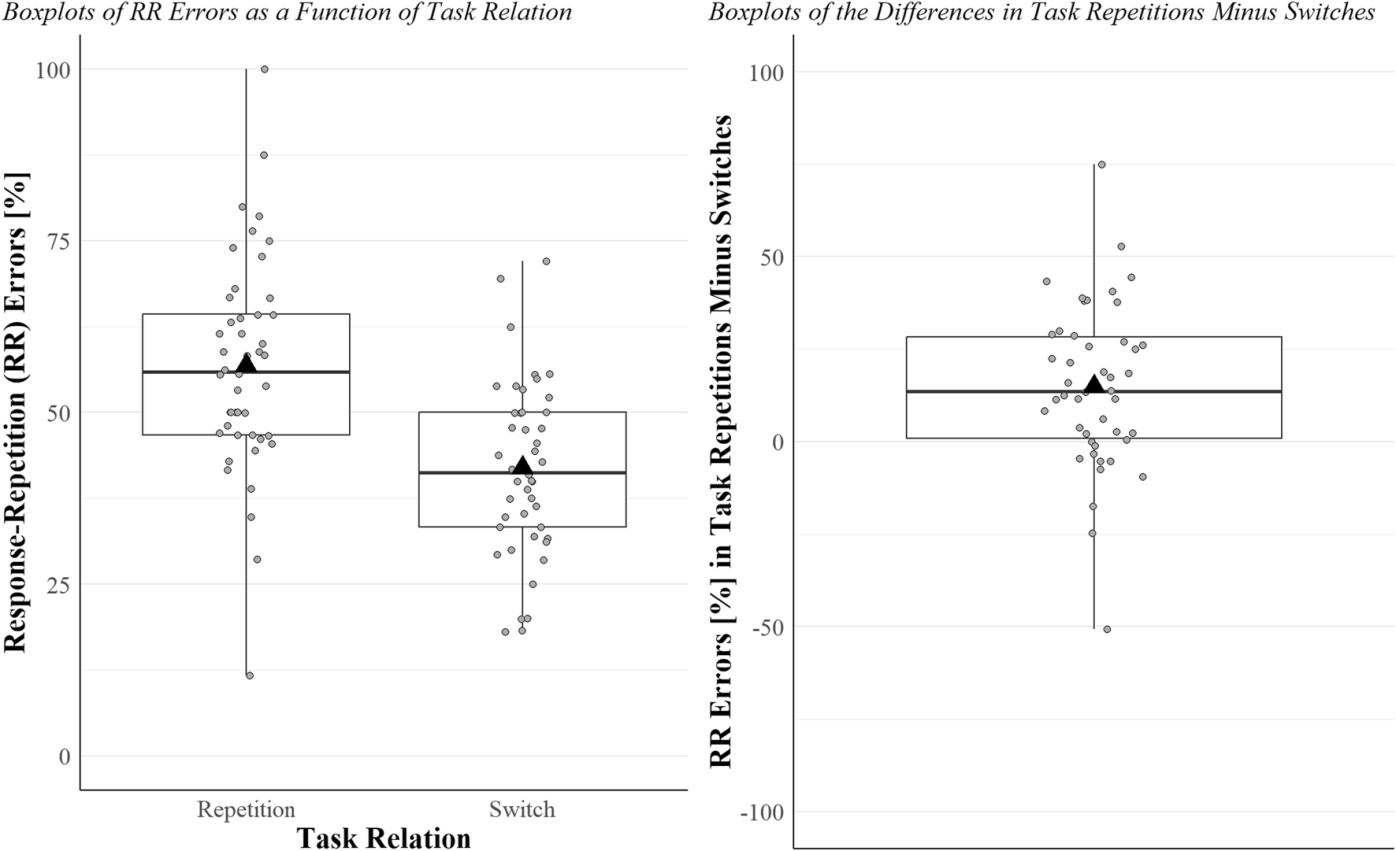
Three boxplots are shown. In each boxplot, the black triangle represents the mean, the thick horizontal line the median. The upper and lower borders of the box represent the third and the second quartiles, respectively, and the upper and lower vertical bars indicate the fourth and the first quartiles, respectively. The left panel shows the percentage of response-repetition errors in task repetitions and in switches for each participant (one participant is one dot in each boxplot). The right panel shows the difference in the percentage of response-repetition errors in task repetitions minus switches for each participant. Dots above the 0 (the majority) represent participants with greater percentage of response-repetition errors in task repetitions than in switches, who thus are consistent with the mean pattern. Percentage of response-repetition errors are calculated over the number of errors in response switches per participant per condition (i.e., task repetition vs. switch)

The one-way ANOVA revealed a significant main effect of task relation, *F*(1, 45) = 21.12, *p* < .001, η_p_^2^ = 0.32, whereby most of the errors were response-repetition errors in task repetitions (56.8%), but not in task switches (41.9%; Fig. 1).^7^

#### Error Rates (ERs) and Correct-Trial Response Times (RTs)

To analyse participants’ ERs, we cleaned the data as in the response-repetition errors ANOVA, but we included RRs, leaving 78.3% of the observations.^8^

The results (Table 1) showed a smaller ER in task repetitions (0.15) than in task switches (0.22), and RR benefits in task repetitions (0.02, *t*(45) = 1.79, *p* = .081, *d*_*z*_ = 0.23) became costs in task switches (-0.06, *t*(45) = −4.38, *p* < .001, *d*_*z*_ = 0.65).

**Table 1:** Result of the ANOVA on ERs and correct-trials RTs in Experiment 1

	Error Rates			Reaction Times		
	*F*(1,45)	${\eta}_{\textrm{p}}^2$	${\eta}_{\textrm{G}}^2$	*F*(1,45)	${\eta}_{\textrm{p}}^2$	${\eta}_{\textrm{G}}^2$
Task Relation	41.4***	0.479	0.136	44.79***	0.499	0.052
Response Relation	2.84	0.059	0.01	22.63***	0.335	0.009
Task Relation x Response Relation	24.21***	0.35	0.052	31.2***	0.409	0.011

Note.***p < .001,***p < .01,*p < .05.

To analyse the RTs, we removed all error trials, leaving 64.1% of the observations. We found (Table 1) faster task repetitions (605 ms) than task switches (657 ms), and RR benefits in task repetitions (45 ms, *t*(45) = 7.08, *p* < .001, *d*_*z*_ = 1.06) disappeared in task switches (-1 ms, *t*(45) = −0.32, *p* = .752, *d*_*z*_ = 0.05). Finally, we found shorter RTs in RRs (620 ms) than in response switches (641 ms).

## Experiment 2

In Experiment 2, we included a manipulation that might modulate task-triggered retrieval: an irrelevant cue dimension (the context, henceforth) that switched or repeated orthogonally to the task and the response in each trial. Previous task-switching studies found that context repetitions augmented RR benefits in task repetitions compared to context switches (Benini et al., 2022, 2023; Koch, Frings et al., 2018). These results suggested that this context is bound with the task and the response, although neither task- nor response-relevant. Consequently, a repeating context might retrieve the bound *n−1* response and task, causing performance benefits when both the task and the response repeat, and costs otherwise (Frings et al., 2020; Hommel et al., 2001).

We expected task repetitions to increase the probability of response-repetition errors, as in Experiment 1. Based on the above-mentioned findings, we expected context repetitions (as compared with context switches) to increase the probability of response-repetition errors when the task repeats, but not when the task switches, leading to an over-additive effect of task relation and context relation. Concerning the ERs and RTs analyses, we expected to replicate the RR effect and larger RR benefits in task repetitions when the context also repeated than when it switched (Benini et al., 2022, 2023; Koch, Frings et al., 2018).

### Method

#### Participants

For Experiment 2, we planned to collect at least double the sample size than for Experiment 1, since we introduced another two-level factor (context relation), which would halve the number of observations per cell and was expected to modulate the RR effect by reducing it in context switches. Due to multiple participants starting the experiment at the same time, 112 participants eventually completed the experiment, and we removed two participants who had an average ER greater than 40% (like in Experiment 1). Here, we considered the interaction as a two-tailed paired-samples *t* test on the difference between the RR effect in context repetition versus switches. With 110 participants, G*Power (Faul et al., 2009) indicates an 80% probability to detect an effect size *d*_*z*_ = 0.27 (η_p_^2^ = 0.07) with alpha = .05. Thus, 110 participants provided enough power to detect an effect roughly four times smaller than what we found in the main analysis of Experiment 1, namely η_p_^2^ = 0.32.

As in Experiment 1, we recruited RWTH students fluent in English, between 18 and 35 years old, and we rewarded them with course credits or payment (5 € for about 35 min). The 110 participants (85 females, none diverse, seven did not declare) were on average 22.2 ± 4.7 years old, and 13 were left-handed (six did not declare).

#### Stimuli, tasks and responses

The stimuli, tasks, cues and response keys were identical to Experiment 1. The difference lay in the addition of context information, such that the cues were shown in black during the instructions, but were otherwise either red or blue during the training and the experimental trials.

#### Procedure

Data collection took place between November 2021 and July 2022.^9^ The procedure was identical to Experiment 1, except for the following differences. The instructions warned participants that the cues would appear either in blue or in red, but this was not relevant to their task. The experimental trials were organized in six blocks of 72 trials. As in Experiment 1, in each block, each digit was presented in all the font sizes, once per task. Furthermore, each cue was presented equally often in blue and red. Finally, in the six blocks overall, each digit in a certain font size was presented exactly three times in a blue context and three times in a red context.

#### Design and analyses

The experiment had a 2 ×2 ×2 within-subject design with the factors *task relation* (repetition vs. switch), *context relation* (repetition vs. switch) and *response relation* (repetition vs. switch). As in Experiment 1, our main hypothesis consisted of a larger probability of response-repetition errors in task repetitions than switches. Furthermore, our second hypothesis was for this difference to be larger in context repetitions than switches. To test these hypotheses, we ran a two-way within-subjects ANOVA with task relation (repetition vs. switch) and context relation (repetition vs. switch) as the independent variables and the probability of response-repetition errors as the dependent variable. This probability was calculated as in Experiment 1, that is, by dividing the number of response-repetition errors in each of the four experimental conditions by the total number of errors in response switches in that condition in the cleaned dataset. We planned to run two one-tailed paired-sample *t* tests to examine whether the probability of response-repetition errors in task repetition was significantly larger than in switches in both context repetitions and context switches.

To examine whether our data pattern replicates the RR effect and the modulation of it by context relation (Benini et al., 2022, 2023; Koch, Frings et al., 2018), we ran two further three-way within-subjects ANOVAs with task relation (repetition vs. switch), response relation (repetition vs. switch) and context relation (repetition vs. switch) as independent variables, and with ERs and correct-trials RTs as the dependent variables, thus including also RR trials.

### Results

#### Probability of RR errors

**Fig. 2:**
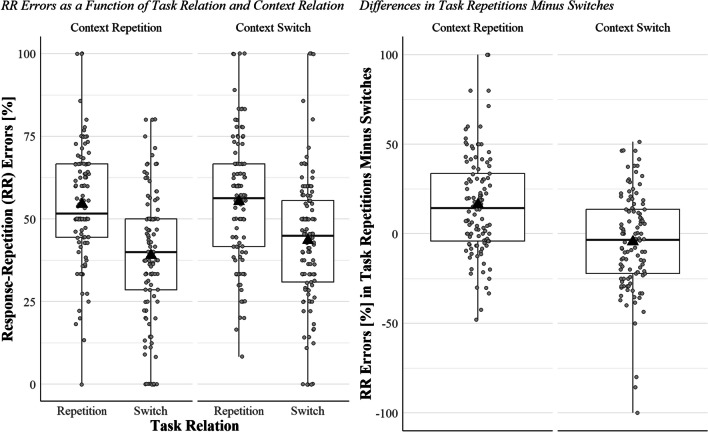
Five boxplots are shown. In each boxplot, the black triangle represents the mean, the thick horizontal line the median. The upper and lower borders of the box represent the third and the second quartiles, respectively, and the upper and lower vertical bars indicate the fourth and the first quartiles, respectively. The left panel shows the percentage of response-repetition errors in task repetitions and in switches divided by context repetitions and switches for each participant (one participant is one dot in each boxplot). The right panel shows the difference in the percentage of response-repetition errors in task repetitions minus switches divided by context repetitions and switches for each participant. Dots above the 0 (the majority in the boxplot showing context repetition trials) represent participants with greater percentage of response-repetition errors in task repetitions than in switche, who thus are consistent with the mean patterns. Percentage of response-repetition errors are calculated over the number of errors in response switches per participant per condition (i.e., task repetition vs. switch by context repetition vs. switch)

From the complete dataset, we removed response repetitions (33.4%), the first trial of each block (1.4%), post-error trials (19.0%), all trials with RT shorter than 200 ms (0.3%) and timed-out trials (1.4%). From the response switches, we thus discarded 20.9% of the original trials. The 79.1% of response switches that we analysed included 83.4% correct responses, 7.9% response-repetition errors, and 8.6% residual errors. The two-way ANOVA revealed a significant main effect of task relation, *F*(1, 109) = 57.23, *p* < .001, η_p_^2^ = 0.34, such that most of the errors were response-repetition errors in task repetitions (54.9%), but not in task switches (41.2%, Fig. 2).^10^ The main effect of context relation was non-significant, *F*(1, 109) = 2.49, *p* = .118, η_p_^2^ = 0.02, so was its interaction with task relation, *F*(1, 109) = 1.28, *p* = .261, η_p_^2^ = 0.01. The planned comparisons showed a larger probability of response-repetition errors in task repetitions than task switches in both context repetitions (+ 15.5%, *t*(109) = 6.60, *p* < .001, *d*_*z*_ = 0.63) and context switches (+ 11.86%, *t*(109) = 4.64, *p* < .001, *d*_*z*_ = 0.44, see Fig. 2).

#### ERs and Correct-Trials RTs

To analyse participants’ ERs, we cleaned the data as in Experiment 1, leaving 79.0% of the observations. The ANOVA (Table 2) indicated fewer errors in task repetitions (0.14) than task switches (0.20), and RR benefits in task repetitions (0.05, *t*(109) = 8.16, *p* < .001, *d*_*z*_ = 0.78) became costs in task switches (-0.05, *t*(109) = −6.89, *p* < .001, *d*_*z*_ = 0.66). We found small overall context repetition costs (-0.02).

**Table 2:** Results of the ANOVA on ERs and correct-trials RTs in Experiment 2

	Error Rates			Reaction Times		
	*F*(1,109)	${\eta}_{\textrm{p}}^2$	${\eta}_{\textrm{G}}^2$	*F*(1,109)	${\eta}_{\textrm{p}}^2$	${\eta}_{\textrm{G}}^2$
Task Relation	92.79***	0.46	0.082	210.78***	0.659	0.05
Response Relation	0.11	<.001	<.001	16.9***	0.134	0.003
Context Relation	18.19***	0.143	0.008	6.82*	0.059	< .001
Task Relation x Response Relation	106.7***	0.495	0.069	148.38***	0.576	0.018
Task Relation x Context Relation	1.9	0.017	0.001	0.72	0.007	<.001
Response Relation x Context Relation	1	0.009	<.001	8.44*	0.072	<.001
Task Relation x Response Relation x Context Relation	0.05	<.001	<.001	0.74	0.007	<.001

Note.***p <.001,** *p <*.01, *p <.05.

To analyse the RTs, we removed all error trials, leaving 65.9% of the observations. The ANOVA (Table 2) indicated that task repetitions (620 ms) were faster than switches (668 ms) and that RR benefits in task repetitions (39 ms, *t*(109) = 11.25, *p* < .001, *d*_*z*_ = 1.08) became RR costs in task switches (-17 ms, *t*(109) = −4.58, *p* < .001, *d*_*z*_ = 0.44).We found shorter RTs in RRs (639 ms) than response switches (650 ms). We found small context repetition *costs* (-5 ms), and smaller RR benefits in context repetitions (5 ms) than in context switches (17 ms).

## General discussion

The present study tested whether the response and the task become bound in each trial by examining the task-triggered retrieval of the *n−1* response. In two task-switching experiments, participants responded by pressing one of three keys. In response switches, we classified each response as either correct, a response-repetition error, or a residual error. If the task indeed becomes bound with the response in each trial, a repeating task should retrieve the previous response (Altmann, 2011; Frings et al., 2020). Thus, the probability of committing response-repetition errors should be higher in task repetitions than in task switches, since a switching task should not retrieve the *n−1* binding (Altmann, 2011; Frings et al., 2020). Intriguingly, the probability of response-repetition errors was indeed larger in task repetitions than in switches in both experiments (Figs. 1 and 2).

### Implications for theoretical accounts of the RR effect

Four main accounts for the RR effect exist in task switching: the response-inhibition and priming account (Grzyb & Hübner, 2013; Hübner & Druey, 2006), the reconfiguration account (Kleinsorge, 1999; Kleinsorge & Heuer, 1999), the associative-learning account (e.g., Rogers & Monsell, 1995; Schuch & Koch, 2004), and the episodic-retrieval accounts (Altmann, 2011; Frings et al., 2020; Hommel et al., 2001). All accounts predict RR benefits in task repetitions and RR costs in task switches (Druey, 2014; Gade et al., 2014). Episodic-retrieval accounts propose that RR benefits in task repetitions derive from the task-triggered retrieval of the *n−1* response, which is also the correct response in RRs. However, in the three remaining accounts, RR benefits hinge on *target-category* repetitions, rather than on task repetitions. Indeed, the response-inhibition and priming account proposes that the repetition of the relevant target category primes the just-executed response; the reconfiguration account proposes that repetition of the (higher-level) target category is necessary for RR benefits to emerge; the associative-learning account maintains that repeating the target category retrieves the most-strongly associated *n−1* response. Those task-switching studies that use two responses per task cannot distinguish between repeating the task and repeating the target category, as they consider trials where both the task (parity) and the response (left) repeat: here, necessarily, the task-relevant target category (even) repeats. In contrast, we analysed *erroneous* RR in response *switches,* which are trials where the relevant target category cannot repeat.^11^ Crucially, participants committed more response-repetition errors when the task repeated, although the target category switched. Episodic-retrieval accounts, postulating a task-triggered retrieval of the *n−1* response, predict this result. The three remaining accounts, postulating reactivation of the response by a repeating target category, cannot predict this result without adding assumptions. Indeed, a switch in the target category (i) *prevents* response re-activation in the response inhibition and priming account, and calls for a response *switch* in both (ii) the reconfiguration account and (iii) the associative-learning account. Our paradigm and analyses thus provided compelling evidence for the existence of a task-triggered retrieval of the *n−1* response. Note though that the existence of retrieval does not rule out the existence of other processes maintained by the alternative accounts. Furthermore, while we could rule out that target-category repetition is necessary to retrieve the *n−1* response, we could not rule out the exact mechanism through which task and response are bound. That is, an associative learning account that postulates strengthening of *task*-response associations in each trial would predict our results as well as a short-term task-response binding account.

Finally, note that since we only used one cue per task, we cannot disentangle whether it is the repeating task that triggers response retrieval or the repeating cue. However, in Experiment 2 the cue did not always repeat identically in task repetitions, but it could be in a different colour due to context switch trials. Since context repetition did not modulate the effect of task repetition, we could tentatively exclude that our effects are due to perceptual cue repetitions alone.

In the present study, we tested whether a repeating task erroneously retrieves the previous response, which can explain RR benefits in task repetitions, while we did not test whether a repeating *response* erroneously retrieves the previous *task*, which could explain RR costs in task switches. The pattern of *residual errors* is also not helpful to uncover retrieval processes in task switches, but, intriguingly, they are consistent with predictions of the three alternative accounts. Indeed, the alternative accounts predict that residual errors should be more likely than response-repetition errors in task switches,^12^ as we found. This result suggests that when the *n−1* response is not retrieved – in task switches – repeating the response is indeed less likely than switching it. Therefore, we conclude that while *n−1* response retrieval may cause RR benefits in task repetitions, response-inhibition or reconfiguration processes may cause RR costs in task switches. In other words, a *hybrid account* (cf. Koch, Frings et al., 2018) including both episodic-retrieval and response-inhibition processes is the one that best explains our data.

### Binding of context features

In Experiment 2, we examined whether an irrelevant context (i.e., the task-cue colour) is bound with relevant features and thus retrieves *n−1* bindings when it repeats (see Benini et al., 2022, 2023; Koch, Frings et al., 2018, for evidence of task-response-context bindings in task switching). If so, repeating the context might augment the task-triggered retrieval of the *n−1* response. The results went in this direction, but only descriptively.

Concerning the RTs and ERs analyses (Table 2), Benini et al. (2022, 2023) and Koch, Frings et al. (2018) found that RR benefits in task repetitions were larger when the context repeated than when it switched. This is consistent with the context being bound with the task and the response and retrieving the *n−1* binding so that performance benefits are observed in full repetitions. We did not replicate this pattern. Surprisingly, RR benefits in task repetitions were larger in context switches than in repetitions. This result is difficult to explain and might be due to the presence of three response options versus two options, as in the cited studies. Since response switches were more likely than repetitions, this maybe counteracted context-triggered RR benefits.

## Conclusion

Across two experiments, the probability of response-repetition errors increased when the task repeated than when it switched, supporting the notion that the task and the response become bound in task switching. This is predicted by episodic-retrieval accounts for the RR effect, while it is not predicted by other competing accounts. The probability of response-repetition errors was not significantly higher when repeating versus switching an irrelevant context, therefore yielding no evidence that the irrelevant context is also bound with the task and the response in this study. In sum, the present results support task-triggered response retrieval as a process responsible for the RR benefits in task repetitions and, therefore, constitutes a novel contribution to the debate about the RR effect.

## Footnotes

1 Such bindings might be formed between the response and the task and/or the response and the target (Altmann, 2011; Frings et al., 2020; Grange et al., 2017; Hommel et al., 2001) or target category (Rogers & Monsell, 1995; Schuch & Koch, 2004) or the task and the target (Koch & Allport, 2006; Waszak et al., 2003). For example, Altmann (2011) suggested that the elements of a trial are bound so that costs are observed in trial *n* whenever only some elements repeat from trial *n−1*. In this work, however, we focus on task-response bindings, since we only consider conditions in which the target and the target category never repeat.

2 In the present work, we highlight the contrasting predictions derived by the account postulating an association between the stimulus category and the response (Rogers & Monsell, 1995) and the episodic-retrieval accounts postulating task-response binding. Specifically, we demonstrate that response-retrieval also takes place in the absence of target-category repetitions. However, we cannot disentangle whether the task and the response become associated via an associative-learning or rather a binding mechanism.

3 We did not use the numbers from 1 to 6 as, in a pilot test, participants very readily categorized 1 as small in value, creating a difference between stimuli and tasks.

4 The font sizes did not increase by the same ratio from the smallest to the largest, rather the sizes belonging to the same category increased by a smaller ratio (e.g., 30 pt and 42 pt) than the sizes belonging to the next category ratio (e.g., 42 pt and 67 pt). We selected these font sizes as they reduced the font-task difficulty in a pilot study, consequently rendering the font task as easy as the value task.

5 Note that, in some trials, committing a response-repetition error coincides with responding to the distractor dimension (e.g., to the font size when the task is value). However, such response-to-the-distractor errors should be more likely in task switches than in task repetitions. Indeed, in task switches, they could reflect a failure to switch the attention to the task-relevant dimensions, while in task repetitions it should be easier to maintain the attention on the same task-relevant dimension as in the previous trial, and thus ignore the distractor. Therefore, such responding-to-the-distractor process does not confound our main prediction, rather it should work against it.

6 Note that the results were the same when the number of response switches, instead of errors in response switches, was taken as the reference (i.e., denominator) to calculate the probability of response-retrieval errors.

7 The pattern holds as well when the response-retrieval error probability and the residual-error probability were not conditioned on committing an error. In other words, when considering each error type in response switches overall, the probability of response-retrieval errors was higher in task repetitions (9.5%) than in task switches (8.0%, *F*(1, 45) = 4.56, *p* = .038, η_p_^2^ = 0.09. Also, residual errors were overall more likely in task switches (11.3%) than repetitions (7.1%), *F*(45) = 21.86, *p* < .001, η_p_^2^ = 0.33. In task switches, residual errors were also more likely than response-retrieval errors, *t*(45) = 3.84, *p* < .001, *d*_*z*_ = 0.57.

8 Note that the results do not change if immediate font sizes and/or digit repetitions from trial *n-1* to *n* are removed from the analyses. The same holds for Experiment 2.

9 In spite of being an online experiment, our data collection took a long time due to our choice of recruiting students only instead of using online recruiting platforms.

10 As in Experiment 1 (see footnote 5), the pattern holds when the different error probabilities were not conditioned on making an error. The probability of response-retrieval errors was higher in task repetitions (9.1%) than task switches (7.3%, *F*(1, 109) = 24.44, *p* < .001, η_p_^2^ = 0.18). Residual errors were overall more likely in task switches (10.1%) than repetitions (7.8%), *F*(109) = 28.20, *p* < .001, η_p_^2^ = 0.21. In task switches, residual errors (10.1%) were also more likely than response-retrieval errors (7.3%), *t*(109) = 6.50, *p* < .001, *d*_*z*_ = 0.62.

11 Indeed, if the response switches and if the target is an even digit in *n−1*, it must be odd in *n* (in task repetitions), or the relevant dimension must have switched so that the digit is smaller or larger than five (in task switches).

12 The response inhibition account maintains that the *n−1* response is inhibited, thus response-retrieval errors should be less likely than residual errors, since the latter are not inhibited. The reconfiguration account makes the same prediction, since a switch at the hierarchically higher task-level demands a switch at the hierarchically lower response-level. The associative learning account as well assumes that, in task switches, the association between the *n−1* response (e.g., left) and the current target category (e.g., even) was weakened in trial *n−1*, where the *n−1* response (e.g., left) got associated with the other category (e.g., greater than five).
